# Medicinal plants and natural compounds against acyclovir-resistant HSV infections

**DOI:** 10.3389/fmicb.2022.1025605

**Published:** 2022-10-10

**Authors:** Lin Xu, Xuan-Lei Zhong, Zhi-Chao Xi, Yang Li, Hong-Xi Xu

**Affiliations:** ^1^School of Pharmacy, Shanghai University of Traditional Chinese Medicine, Shanghai, China; ^2^Engineering Research Center of Shanghai Colleges for TCM New Drug Discovery, Shanghai, China; ^3^Shuguang Hospital, Shanghai University of Traditional Chinese Medicine, Shanghai, China

**Keywords:** herpes simplex virus, antiviral herbs, natural products, acyclovir-resistant, mode of action

## Abstract

Herpes simplex virus (HSV), an alphaherpesvirus, is highly prevalent in the human population and is known to cause oral and genital herpes and various complications. Represented by acyclovir (ACV), nucleoside analogs have been the main clinical treatment against HSV infection thus far. However, due to prolonged and excessive use, HSV has developed ACV-resistant strains. Therefore, effective treatment against ACV-resistant HSV strains is urgently needed. In this review, we summarized the plant extracts and natural compounds that inhibited ACV-resistant HSV infection and their mechanism of action.

## Introduction

**Figure 1:**
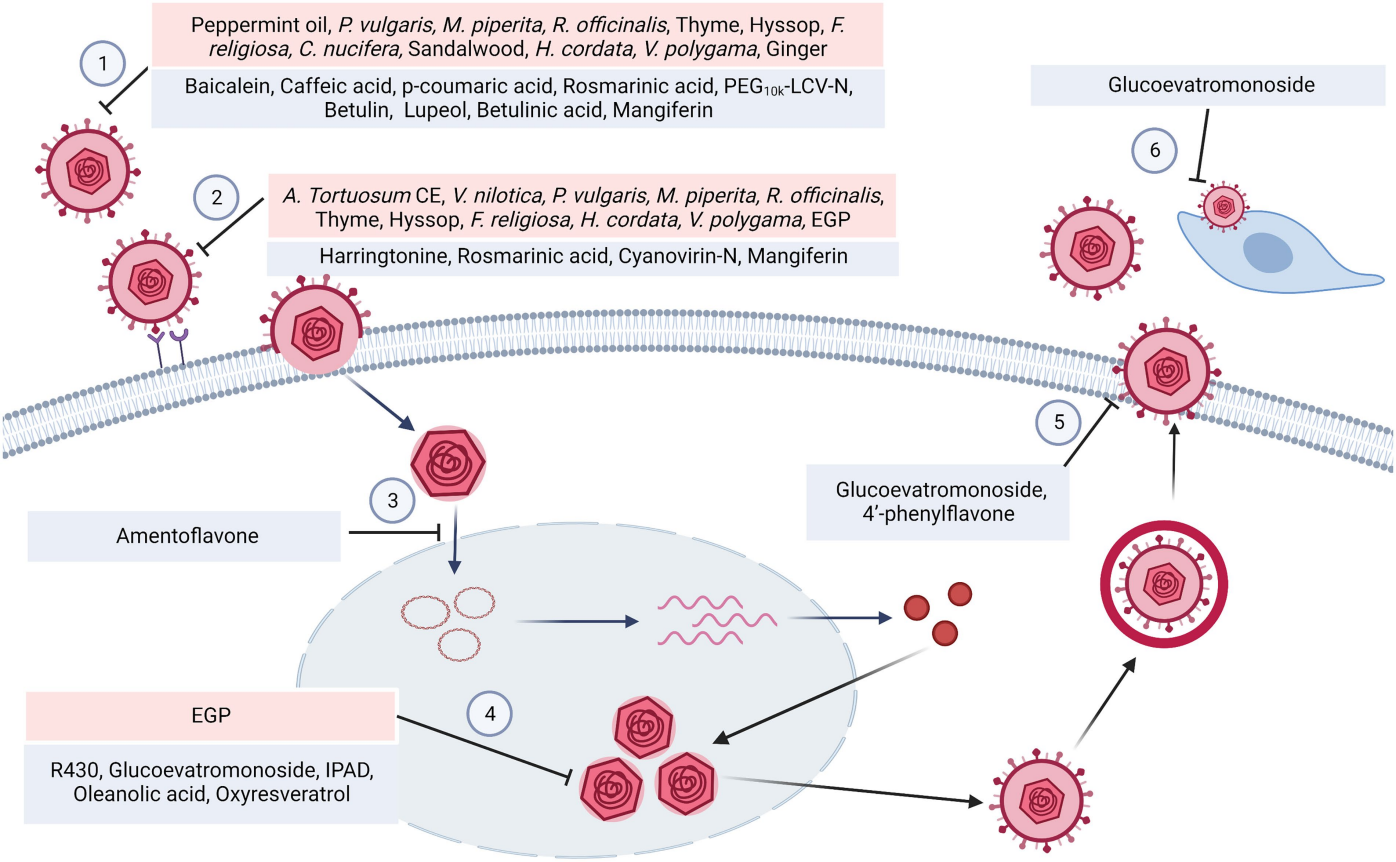
HSV replication cycle and the steps that were targeted by the phytomedicines mentioned in this review. (1) Plant extracts such as peppermint oil, *P. vulgaris* extract as well as the phytochemicals such as baicalein, caffeic acid showed virucidal effect. (2) HSV virions requires a set of viral glycoproteins to mediate host cell attachment and entry, which could be suppressed by *V. nilotica* extract, *M. piperita* extract, harringtonine and etc. (3) Then the capsids are transported into the nucleus, which could be inhibited by Amentoflavone. (4) The biosynthesis process produced viral DNA, which could be blocked by amentoflavone, R430 and IPAD. The phytomedicines suppressed the helicase-primase complex, Immediate Early genes (ICP0, ICP4, ICP27), Early genes (ICP6, ICP8) and Late genes (UL19, UL27, UL44, US6). (5) Glucoevatromonoside and 4′-phenylflavone exerted the inhibitory effect on virion egression. (6) The cell-to-cell spread could be interfered by glucoevatromonoside.

Herpes simplex virus (HSV) is an alphaherpesvirus belonging to the Herpesviridae family. The virion is made up of large double-stranded DNA, an icosapentahedral capsid, a tegument, and a glycoprotein-filled envelope (Whitley and Roizman, 2001). The viral entry into the host cells begins with the interaction between viral glycoproteins, gB, and gC with cell surface receptors. The fusion of the viral envelope is mediated by glycoproteins, gD, gB, and the heterodimer gH/gL, allowing the viral entry into the host cells (Kukhanova et al., 2014). After the entry of the viral particle, the capsid is trafficked to the nuclear pores along a network of microtubules and subsequently into the nucleus (Mues et al., 2015). VP16 triggers the transcription of six immediate early (IE) genes (ICP0, ICP4, ICP22, ICP27, ICP47, and US1.5) and the activation of early (E) and late (L) genes. With the help of the helicase-primase complex and some viral proteins, such as UL9 and ICP8, the viral DNA unwinds and replicates. The capsid is initially assembled in the nucleus and then transported into the cytoplasm. The virion is trafficked intracellularly, during which the virion forms an outer shell, package, and assemble. Finally, the virion is egressed to infect other cells, resulting in cell-to-cell spread. The replication cycle lasts for 18–20 h, which has been briefly illustrated in Figure 1. After primary infection, HSV virions replicate in large numbers in neuronal cells, enter sensory neuron axons, and hide in the neuronal cell nucleus (Kukhanova et al., 2014). The viruses establish latent infections, which are reactivated by persistent psychological stress and other stimuli (Cohen et al., 1999). HSV infections have become a major public health concern. According to a WHO investigation, more than one billion people worldwide are infected with HSV-1-caused oral herpes, and an estimated 500 million are infected with HSV-2-caused genital herpes (Kushch et al., 2021).

Acyclovir (ACV) is widely used to treat HSV infections and inhibits HSV-specific DNA polymerase and impedes HSV replication and further infection (Brigden and Whiteman, 1983). Since HSV infection is incurable and recurrent and prolonged, the excessive use of nucleoside analogs such as ACV causes severe side effects, including neurotoxicity and renal impairment (Paluch et al., 2021) and the emergence of drug resistance. ACV resistance was reported to be 7% in immunocompromised patients, but was only 0.27% in healthy immunocompetent adults. The emergence of drug-resistant HSV strains restricts therapeutic options, preventing timely treatment and causing a variety of diseases (Stranska et al., 2005). The mutant of HSV-induced TK led to ACV resistance in 95% of cases, and the mutant of DNA polymerase (DNA-pol) enzymes also accounted for resistance (Field, 2001; Morfin and Thouvenot, 2003). The resources and mechanism of resistance of ACV-resistant HSV strains mentioned in this review were organized in Table 1.

**Table 1:** Review of the ACV-resistant strains used in the collected literature.

	Strain	Resource	Mechanism of resistance	Ref
HSV-1 ACVr	ACGr4	Cultured in lab	TK deficient	Parris et al., 1978; Priengprom et al., 2015
	dlsptk	Genetically engineered	TK deletion	Valyi-Nagy et al., 1994; Priengprom et al., 2015
	dxplll	Not mentioned	Phosphonoacetic acid and phosphonoformate resistant	Priengprom et al., 2015
	AR-29	Not mentioned	Not mentioned	Rechenchoski et al., 2020
	R-100	Cultured in lab	TK enzyme encoding gene mutant	Palu et al., 1988; Vilhelmova-Ilieva et al., 2014
	29-R	Not mentioned	Not mentioned	Bertol et al., 2011; Petronilho et al., 2012; Argenta et al., 2015
	106	Clinical isolate	TK mutant	Wang et al., 2011; Jin et al., 2015; Lei et al., 2019; Li et al., 2019; Wang et al., 2020; Shan et al., 2021
	153	Clinical isolate	TK mutant	Wang et al., 2011; Jin et al., 2015; Lei et al., 2019; Li et al., 2019; Wang et al., 2020; Liu Y. et al., 2021; Shan et al., 2021
	Blue	Cultured in lab	TK deletion	Jin et al., 2015; Li et al., 2019; Luo et al., 2020; Wang et al., 2020; Liu Y. et al., 2021; Shan et al., 2021
	A4-3	Cultured in lab	TK deletion	Hayashi et al., 1997; Hayashi et al., 2012
	PAAr	Cultured in lab	DNA polymerase mutant	Honess and Watson, 1977; Jofre et al., 1977; Bourinbaiar and Lee-Huang, 1996; Yoshida et al., 1996; Xu et al., 1999; D'Aiuto et al., 2018
	DM21	Genetically engineered	TK deficient	Efstathiou et al., 1989; Bourinbaiar and Lee-Huang, 1996; Xu et al., 1999; Chiu et al., 2004
	Angelotti	Cultured in lab	DNA polymerase gene mutant	Ott et al., 1979; Schnitzler et al., 2007; Reichling et al., 2008
	1246/99	Patient isolate	TK enzyme encoding gene mutant	Schnitzler et al., 2007; Reichling et al., 2008
	492/02	Patient isolate	DNA polymerase gene mutant	Schnitzler et al., 2007
	B2006	Cultured in lab	TK deficient	Dubbs and Kit, 1964; Yoshida et al., 1996; Ghosh et al., 2004; Chuanasa et al., 2008
	Field	Not mentioned	TK deficient	Ghosh et al., 2004
	AR	Cultured in lab	TK mutant	Su et al., 2008; Chen et al., 2011
HSV-2 ACVr	PU	Not mentioned	TK deficient	Vilhelmova-Ilieva et al., 2014
	Kost	Patient isolate	TK altered	Kost et al., 1993; Bourinbaiar and Lee-Huang, 1996; Xu et al., 1999
	8,708	Patient isolate	TK altered	Kost et al., 1993; Bourinbaiar and Lee-Huang, 1996

This review summarizes a number of ACV-resistant HSV strains that have been genetically engineered or isolated from patients (Table 1), medicinal extracts (Table 2), and phytochemicals (Table 3) that exert antiviral effects and mechanisms of action.

**Table 2:** Review of the plants that show anti-herpes simplex virus activities with their prospective family, part, type of extract, and mode of action.

Family	Plant	Part	Extract	Results	Mode of action	Ref
Araceae	*Arisaema Tortuosum*	Leaves	Chloroform	Vero Cells CC_50_ = 402 μg/ml HSV-2 ACVr EC_50_ = 0.86 μg/ml SI = 467.4	Inhibiting viral attachment and entry as well as the late events of the HSV-2 replication cycle	Ritta et al., 2020
Asteraceae	*Echinacea purpurea*	Aerial parts	Not mentioned	HFF Cells CC_50_ = 1:2 dilution Five ACV-R strains of HSV-1 Median ED_50_ = 1:100 Ten ACV-R strains of HSV-2 Median ED_50_ = 1:200	Probable inhibition of postinfection steps	Thompson, 1998
Caulerpaceae	*Caulerpa racemosa*	Not mentioned	Hot water	Vero Cells CC_50_ > 1,000 μg/ml HSV-1 ACVr B2006 EC_50_ = 2.4 ± 0.7 μg/ml, SI > 417 HSV-1 ACVr Field EC_50_ = 2.2 ± 0.1 μg/ml, SI > 454	Not mentioned	Ghosh et al., 2004
Cecropiaceae	*Cecropia glaziovii* Snethl.	Leaves	Ethanol	Vero Cells CC_50_ > 2.000 μg/ml HSV-1 ACVr 29R EC_50_ = 40 μg/ml, SI = 50	Not mentioned	Petronilho et al., 2012
Fabaceae	*Vachellia nilotica*	Bark	Methanol	Vero Cells CC_50_ = 144 μg/ml HSV-2 ACVr EC_50_ = 6.71 μg/ml, SI = 21.5	Partial virus inactivation; Inhibition of viral attachment	Donalisio et al., 2018
Lamiaceae	*Mentha piperita* L.	Leaves	Not mentioned	RC-37 Cells TC_50_ = 0.014%	Virucidal effect	Schuhmacher et al., 2003
	*Prunella vulgaris*	Dried herbs	Aqueous ethanol	RC-37 Cells Prunella 20% ethanol TC_50_ = 362.4 μg/ml HSV-1 ACVr Angelotti infectivity 1.3% HSV-1 ACVr 1246/99 infectivity 0% Prunella 80% ethanol TC_50_ = 192.1 μg/ml HSV-1 ACVr Angelotti infectivity 0% HSV-1 ACVr 1246/99 infectivity 0%	Virucidal effect; Inhibition of viral adsorption	Reichling et al., 2008
	*Mentha piperita*	Dried leaves	Aqueous ethanol	RC-37 Cells Peppermint 20% ethanol TC_50_ = 422.4 μg/ml HSV-1 ACVr Angelotti infectivity 0% HSV-1 ACVr 1246/99 infectivity 2.4% Peppermint 80% ethanol TC_50_ = 281.6 μg/ml HSV-1 ACVr Angelotti infectivity 0% HSV-1 ACVr 1246/99 infectivity 0%		
	*Rosmarinus officinalis*	Dried leaves	Aqueous ethanol	RC-37 Cells Rosemary 20% ethanol TC_50_ = 253.8 μg/ml HSV-1 ACVr Angelotti infectivity 29.7% HSV-1 ACVr 1246/99 infectivity 0% Rosemary 80% ethanol TC_50_ = 73.9 μg/ml HSV-1 ACVr Angelotti infectivity 0% HSV-1 ACVr 1246/99 infectivity 0%		
	*Thymus vulgaris*	Dried herbs	Aqueous ethanol	RC-37 Cells Thyme 20% ethanol TC_50_ = 293.7 μg/ml HSV-1 ACVr Angelotti infectivity 2.2% HSV-1 ACVr 1246/99 infectivity 0% Thyme 80% ethanol TC_50_ = 62.3 μg/ml HSV-1 ACVr Angelotti infectivity 0% HSV-1 ACVr 1246/99 infectivity 0%		
	*Thymus vulgari* (Thyme)	Essential oil	Not mentioned	RC-37 Cells CC_50_ = 0.007 ± 0.0003% HSV-1 ACVr Aneglotti Infectivity = 4.1 ± 3.2% HSV-1 ACVr 1246/99 Infectivity = 1.2 ± 0.9% HSV-1 ACVr 496/02 Infectivity = 0.3 ± 0.3%	Virucidal effect	Schnitzler et al., 2007
	*Hyssopus officinalis* (Hyssop)	Essential oil	Not mentioned	RC-37 Cells CC_50_ = 0.0075 ± 0.002% HSV-1 ACVr Aneglotti Infectivity = 0.2 ± 0.2% HSV-1 ACVr 1246/99 Infectivity =0.3 ± 0.3% HSV-1 ACVr 496/02 Infectivity =0.1 ± 0.1%		
	*Salvia desoleana* Atzei & V. Picci	SD1(EO fraction)	Not mentioned	Vero Cells CC_50_ = 58.11 μg/ml HSV-2 ACVr EC_50_ = 6.58 μg/ml, SI = 8.83	Inhibition of later step of viral replication cycle	Cagno et al., 2017
	*Prunella vulgaris* L.	Dried spikes	Water	Vero Cells HSV-1 ACVr DM2.1 EC_50_ = 25 ~ 50 μg/ml	Not mentioned	Chiu et al., 2004
			Water-ethanol	Vero Cells CC_50_ > 500 μg/ml	Inhibition of adsorption and penetration	Xu et al., 1999
Moraceae	*Ficus religiosa* L.	Bark	Water	Vero Cells CC_50_ = 1,530 μg/ml HSV-2 ACVr EC_50_ = 6.28 ± 0.04 μg/ml, SI = 243.6	Virucidal effect	Ghosh et al., 2016
			Chloroform	Vero Cells CC_50_ = 809.6 μg/ml HSV-2 ACVr EC_50_ = 13.50 ± 0.50 μg/ml, SI = 59.97	Inhibition of viral attachment and entry; Limitation of viral progeny production	
Palmae	*Cocos nucifera* Linn.	Husk fiber	Water	Crude extract: HEp-2 Cells MNTC = 100 μg/ml HSV-1 ACVr VI = 1.81, PI = 98.4, VII = 3.2, PI>99.9 Vero Cells MNTC = 100 μg/ml HSV-1 ACVr VI = 3, PI>99.9, VII = 3.13, PI>99.9 Fraction II: HEp-2 Cells MNTC = 25 μg/ml HSV-1 ACVr VI = 1, PI = 90.0, VII = 5.0, PI>99.9 Vero Cells MNTC = 100 μg/ml HSV-1 ACVr VI = 3.25, PI>99.9, VII = 4.59, PI>99.9	Virucidal effect	Esquenazi et al., 2002
Rubiaceae	*Nauclea latifolia* Smith	Root bark	CH_2_Cl_2_/MeOH (50:50) mixture	Vero Cells CC_50_ > 100 μg/ml HSV-2 ACVr IC_50_ = 5.38 μg/ml	Inhibition of post-infection stage	Donalisio et al., 2013
Santalaceae	*Santalum album* (Sandalwood)	Essential oil	Not mentioned	RC-37 Cells CC_50_ = 0.0015 ± 0.0001% HSV-1 ACVr Aneglotti Infectivity = 0.2 ± 0.2% HSV-1 ACVr 1246/99 Infectivity =1.1 ± 0.8% HSV-1 ACVr 496/02 Infectivity =0.3 ± 0.2%	Virucidal effect	Schnitzler et al., 2007
Saururaceae	*Houttuynia cordata*	Not mentioned	Water	Vero Cells CC_50_ > 100 mg/ml HSV-1 ACVr AR EC_50_ = 1.11 mg/ml SI = 90.09	Virucidal effect; Inhibition of virus entry (target gD); Inhibition of HSV-induced NF-κB activation	Hung et al., 2015
Verbenaceae	*Lippia Graveolens*	Essential oil	Not mentioned	HEp-2 Cells CC_50_ = 735 μg/ml HSV-1 ACVr EC_50_ = 55.9 μg/ml, SI_50_ = 13.1	Not mentioned	Pilau et al., 2011
	*Vitex polygama* Cham.	Fruits	Ethyl acetate	HEp-2 Cells MNTC = 50 μg/ml HSV-1 ACVr VII = 0.83, PI = 85.2	Virucidal effect; Slight intracellular inhibition	Goncalves et al., 2001
		Leaves		HEp-2 Cells MNTC = 25 μg/ml HSV-1 ACVr VII = 0.58, PI = 73.7	Intracellular inhibition	
		VPAF-1		HEp-2 Cells MNTC = 100 μg/ml HSV-1 ACVr VII = 0.91, PI = 87.7	Inhibition of viral attachment	
Zingiberaceae	*Zingiber officinale* (Ginger)	Essential oil	Not mentioned	RC-37 cells CC_50_ = 0.004 ± 0.001% HSV-1 ACVr Angelotti infectivity 0.2 ± 0.1%, HSV-1 ACVr 1246/99 infectivity 0.3 ± 0.2%, HSV-1 ACVr 496/02 infectivity 0.1 ± 0.1%	Virucidal effect	Schnitzler et al., 2007

**Table 3:** A review of the bioactive natural products reported to have potent anti-HSV properties.

Chemical class	Compound	Structure	Results	**Mode of action**	**Ref**
Alkaloid	Caffeine	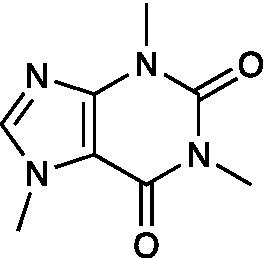	HSV-1 ACVr TK-deficient EC_50_ = 1.10 ± 0.14 mg/ml HSV-1 PAAr EC_50_ = 1.11 ± 0.08 mg/ml	Not mentioned	Yoshida et al., 1996
	Harringtonine	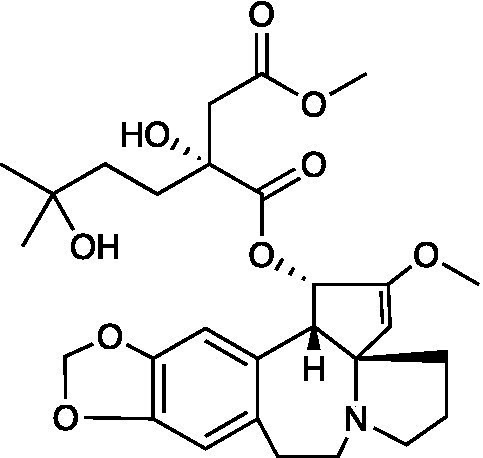	Vero Cells CC_50_ = 239.6 ± 26.3 μM HSV-1 ACVr 153 IC_50_ = 0.1584 ± 0.009 μM, SI = 1512.63 HSV-1 ACVr Blue IC_50_ = 0.1320 ± 0.007 μM, SI = 1815.15	Inhibition of viral membrane fusion and virus entry targeting HVEM	Liu Y. et al., 2021
	R430	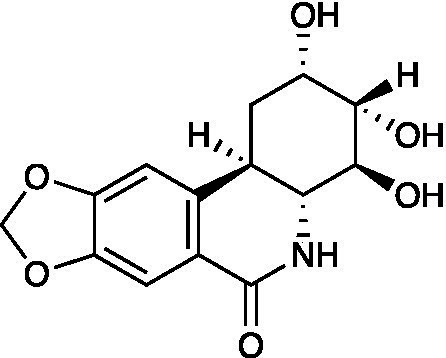	HiPSC Cells CC_50_ = 7.39 μM HSV-1 ACVr TK-deletion EC_50_ = 0.71 μM HSV-1 ACVr PAAr IC_50_ = 0.95 μM	Inhibition of transcription and translation of the viral IE gene ICP4 and DNA polymerase	McNulty et al., 2016; D'Aiuto et al., 2018
Cardenolide	Glucoevatromonoside	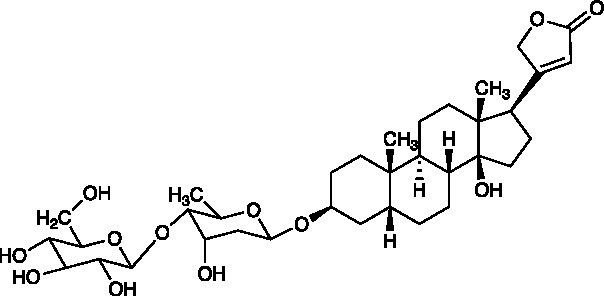	Vero Cells CC_50_ = 273.95 ± 46.46 μM GMK-AH1 Cells CC_50_ > 250 μM HSV-1 ACVr 29R IC_50_ = 0.06 ± 0.01 μM, SI = 4.566	Inhibition of viral protein synthesis (ICP27, UL42, gB, and gD); Reduction of viral cell-to-cell spread	Bertol et al., 2011
Cyclic pentapeptide	*Aspergillipeptide* D	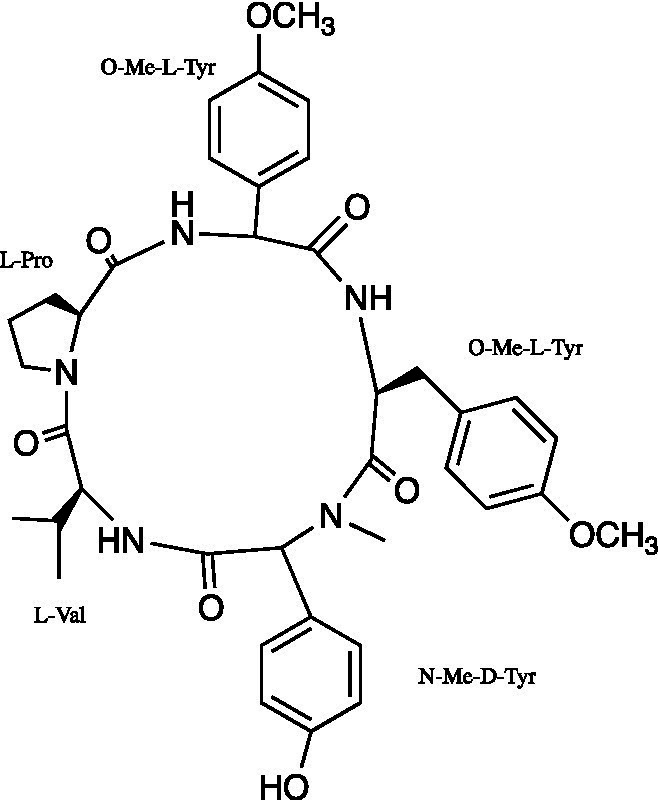	Vero Cells CC_50_ = 208.723 ± 9.717 μM HSV-1 ACVr 106 EC_50_ = 10.486 ± 0.929 μM HSV-1 ACVr 153 EC_50_ = 8.277 ± 1.249 μM HSV-1 ACVr Blue EC_50_ = 7.9875 ± 0.616 μM	Inhibition of intercellular spread targeting gB	Wang et al., 2020
Flavonoid	Amentoflavone	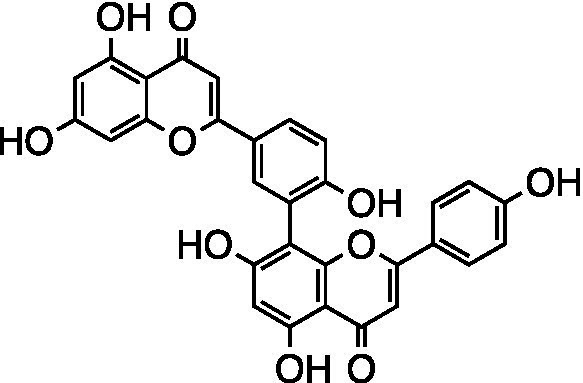	Vero Cells CC_50_ > 100 μM HSV-1 ACVr 106 EC_50_ = 11.11 ± 0.71 μM HSV-1 ACVr 153 EC_50_ = 28.22 ± 2.51 μM HSV-1 ACVr Blue EC_50_ = 25.71 ± 3.97 μM	Inhibition of early viral infection; Reduction of viral nuclear transport by inhibiting cofilin-mediated F-actin assembly	Li et al., 2019
	Baicalein	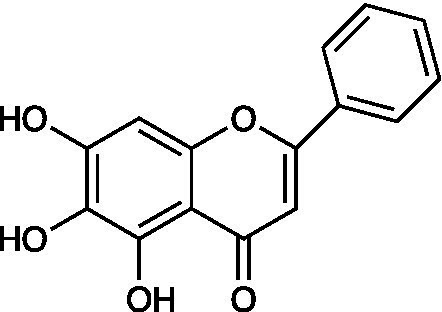	Vero Cells CC_50_ > 200 μmol/l HSV-1 ACVr Blue EC_50_ = 18.6 μmol/l, SI > 10.8 Hacat Cells CC_50_ > 200 μmol/l HSV-1 ACVr Blue EC_50_ = 14.8 μmol/l, SI > 13.5	Inactivation of viral particles; Suppression of NF-κB activation	Luo et al., 2020
	4′-phenylflavone	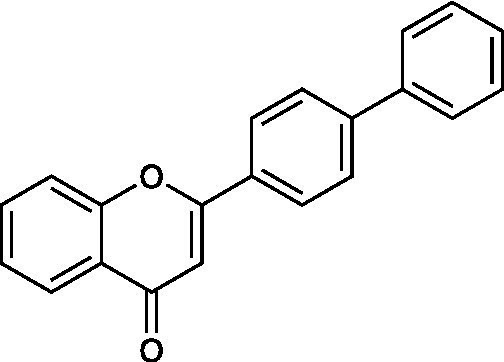	Vero Cells CC_50_ = 510 ± 46 μg/ml HSV-1 ACVr A4-3 IC_50_ = 15 ± 1.4 μg/ml	Inhibition of a late step of viral replication; Reduction of the release of progeny viruses	Hayashi et al., 2012
	Genistein	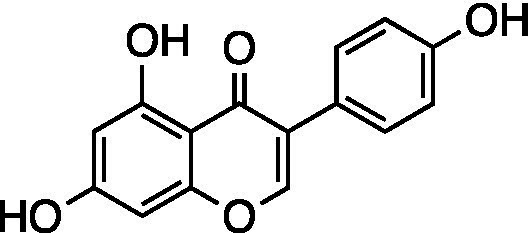	Vero Cells CC_50_ = 54.41 ± 5.39 μM GMK-AH1 Cells CC_50_ = 98.17 ± 14.78 μM HSV-1 ACVr 29R IC_50_ = 7.76 ± 0.76 μM, SI = 7.01	Reduce HSV-1 protein expression	Argenta et al., 2015
	Coumestrol	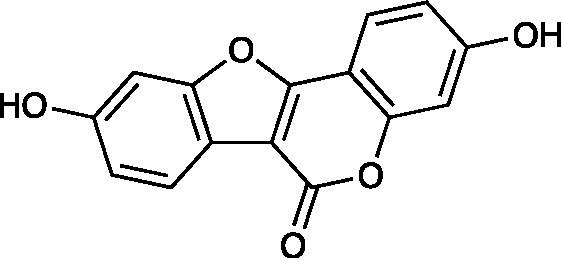	Vero Cells CC_50_ = 105.3 ± 22.33 μM GMK-AH1 Cells CC_50_ > 1,000 μM HSV-1 ACVr 29R IC_50_ = 3.34 ± 0.68 μM, SI = 31.52	Inhibition of the early stages of viral infection; Reduction of HSV-1 protein expression	
Phenolics	Carvacrol	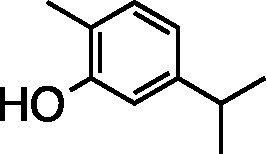	HEp-2 Cells CC_50_ = 250 μg/ml HSV-1 ACVr EC_50_ = 28.6 μg/ml, SI_50_ = 8.7	Inhibition of postinfection stage	Pilau et al., 2011
	Caffeic acid	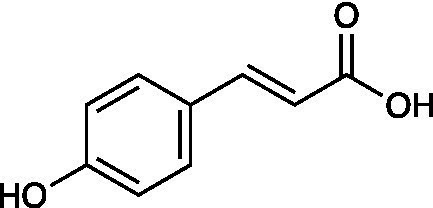	RC-37 Cells CC_50_ = 150 μg/ml HSV-1 ACVr 1 IC_50_ = 100 μg/ml, SI = 1.5 HSV-1 ACVr 2 IC_50_ = 90 μg/ml, SI = 1.7	Virucidal effect	Astani et al., 2014
	*p*-coumaric acid	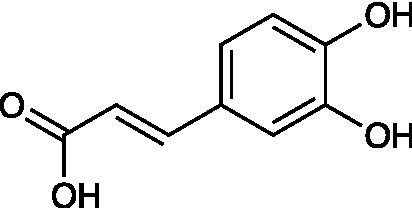	RC-37 Cells CC_50_ ≥ 1,000 μg/ml HSV-1 ACVr 1 IC_50_ = 7 μg/ml, SI ≥ 143 HSV-1 ACVr 2 IC_50_ = 10 μg/ml, SI ≥ 100	Virucidal effect	
	Rosmarinic acid	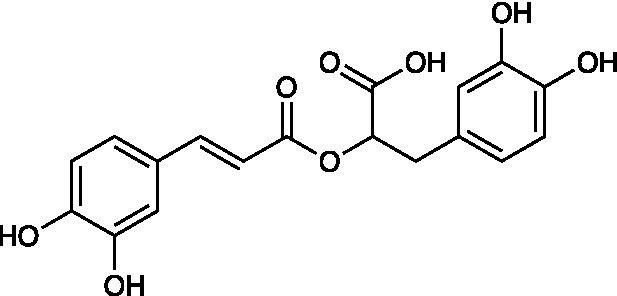	RC-37 Cells CC_50_ = 200 μg/ml HSV-1 ACVr 1 IC_50_ = 10 μg/ml, SI = 20 HSV-1 ACVr 2 IC_50_ = 8 μg/ml, SI = 25	Virucidal effect; Inhibition of viral attachment	
Polyphenolic	Castalagin	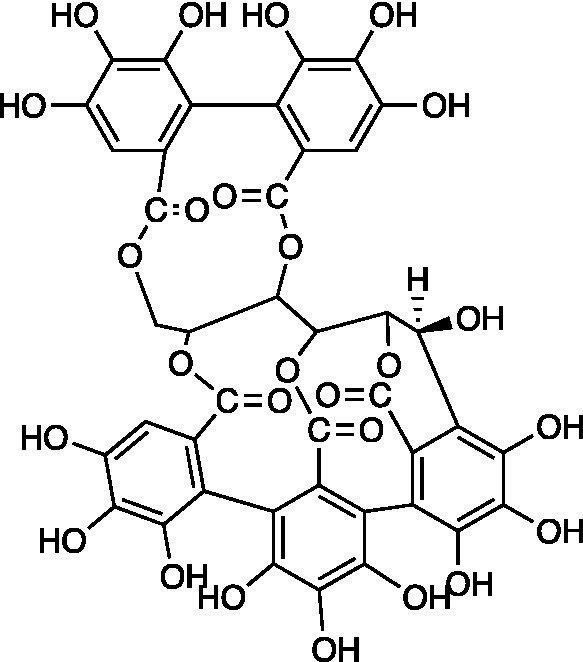	MDBK Cells HSV-1 ACVr R-100 IC_50_ = 0.04 ± 0.002 μM HSV-2 ACVr PU IC_50_ = 0.43 ± 0.03 μM	Not mentioned	Vilhelmova-Ilieva et al., 2014
	Vescalagin	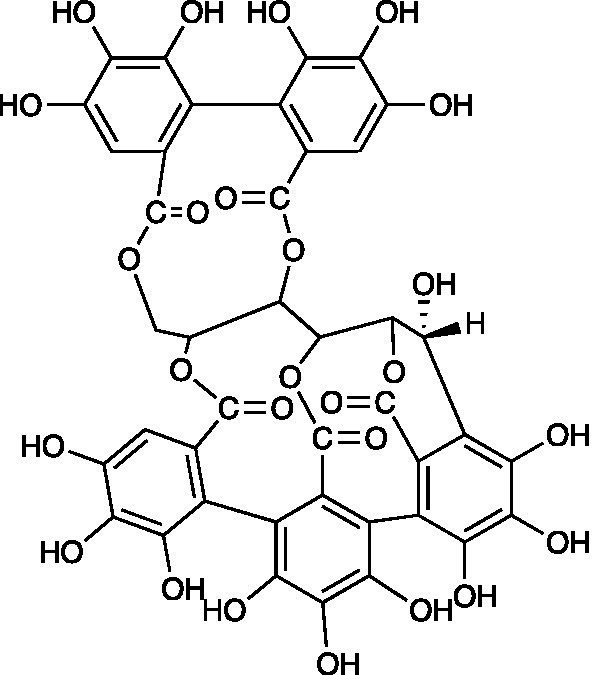	MDBK Cells HSV-1 ACVr R-100 IC_50_ = 0.06 ± 0.003 μM HSV-2 ACVr PU IC_50_ = 0.46 ± 0.0.02 μM		
	Grandinin	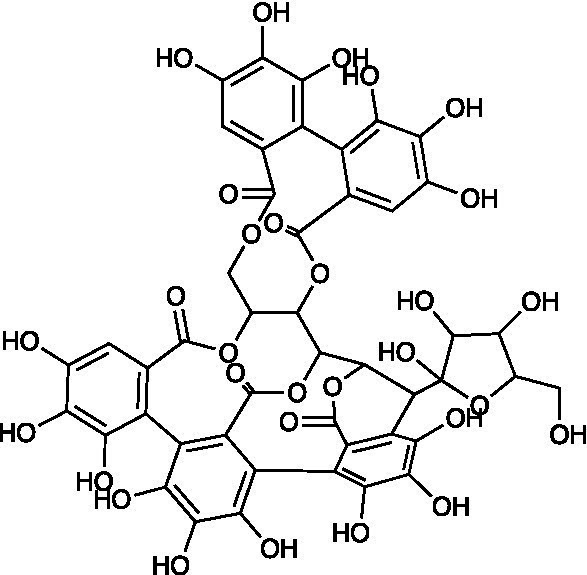	MDBK Cells HSV-1 ACVr R-100 IC_50_ = 0.04 ± 0.02 μM HSV-2 ACVr PU IC_50_ = 0.29 ± 0.02 μM		
Protein	MAP 30	Not mentioned	No detectable toxic effect on WI-38 Cells HSV-1 ACVr DM21 EC_50_ = 0.1 μM HSV-1 ACVr PAAr EC_50_ = 0.1 μM HSV-2 ACVr Kost EC_50_ = 0.3 μM HSV-2 ACVr 8,708 EC_50_ = 0.3 μM	Not mentioned	Bourinbaiar and Lee-Huang, 1996
	GAP31	Not mentioned	No detectable toxic effect on WI-38 Cells HSV-1 ACVr DM21 EC_50_ = 0.5 μM HSV-1 ACVr PAAr EC_50_ = 0.5 μM HSV-2 ACVr Kost EC_50_ = 0.6 μM HSV-2 ACVr 8,708 EC_50_ = 0.8 μM		
	Cyanovirin-N	Not mentioned	Vero Cells CC_50_ = 1.456 ± 0.340 μM HSV-1 ACVr 153 IC_50_ = 0.014 ± 0.002 μM, SI = 104.00 HSV-1 ACVr Blue IC_50_ = 0.010 ± 0.003 μM, SI = 145.60 HSV-1 ACVr 106 IC_50_ = 0.030 ± 0.013 μM, SI = 48.53	Inhibition of viral entry	Lei et al., 2019
	LCV-N	Not mentioned	Vero Cells CC_50_ = 1.747 ± 0.097 μM HSV-1 ACVr 153 IC_50_ = 0.007 ± 0.001 μM, SI = 249.57 HSV-1 ACVr Blue IC_50_ = 0.005 ± 0.001 μM, SI = 349.40 HSV-1 ACVr 106 IC_50_ = 0.022 ± 0.003 μM, SI = 79.41	Not detected	
	PEG_10k_-LCV-N	Not mentioned	Vero Cells CC_50_ = 9.48 ± 1.403 μM HSV-1 ACVr 153 IC_50_ = 0.181 ± 0.047 μM, SI = 52.30 HSV-1 ACVr Blue IC_50_ = 0.218 ± 0.008 μM, SI = 43.48 HSV-1 ACVr 106 IC_50_ = 0.642 ± 0.028 μM, SI = 14.76	Virucidal activity	
Pentacyclic triterpenoid	Oleanolic acid	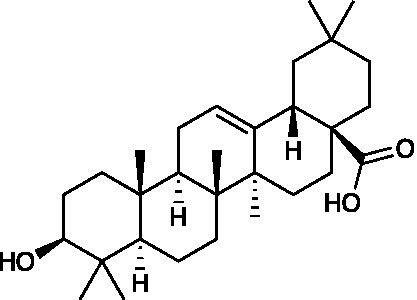	Vero Cells CC_50_ = 39.05 ± 0.561 μM SH-SY5Y Cells CC_50_ = 20.5 ± 0.325 μM HaCaT Cells CC_50_ = 37.06 ± 0.401 μM HSV-1 ACVr 153 EC_50_ = 13.06 ± 0.512 μM HSV-1 ACVr Blue EC_50_ = 13.09 ± 0.642 μM HSV-1 ACVr 106 EC_50_ = 12.89 ± 0.681 μM	Inhibition of the immediate early stage of infection targeting UL8	Shan et al., 2021
Polysaccharide	EGP	Not mentioned	Vero Cells CC_50_ > 1,000 μg/ml HSV-1 ACVr 153 EC_50_ = 1.24 ± 0.32 μg/ml HSV-1 ACVr Blue EC_50_ = 1.48 ± 0.31 μg/ml HSV-1 ACVr 106 EC_50_ = 1.11 ± 0.27 μg/ml	Inhibition of the early stages of viral infection and viral biosynthesis	Jin et al., 2015
Terpene	Betulin	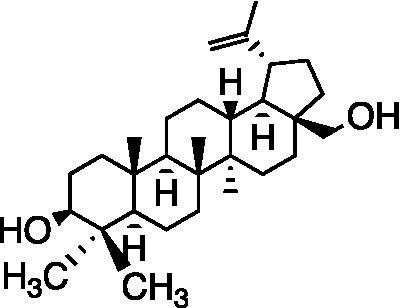	RC-37 Cells CC_50_ = 22 μg/ml	Virucidal effect	Heidary Navid et al., 2014
	Lupeol	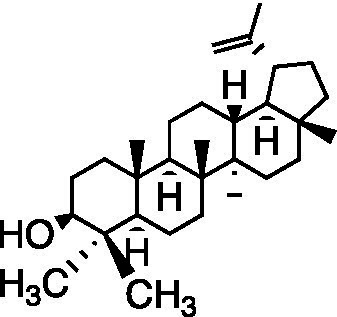	RC-37 Cells CC_50_ = 5 μg/ml		
	Betulinic acid	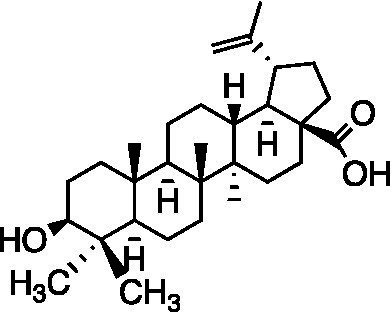	RC-37 Cells CC_50_ = 5 μg/ml		
	IPAD	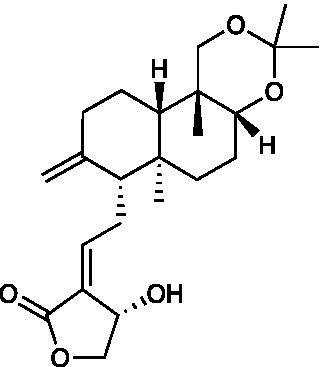	Vero Cells CC_50_ = 39.71 μM HSV-1 ACVr ACGr4 IC_50_ = 17.89 μM, SI = 2.22 HSV-1 ACVr dlsptk IC_50_ = 16.86 μM, SI = 2.36 HSV-1 ACVr dxplll IC_50_ = 17.12 μM, SI = 2.32	Inhibition of viral biosynthesis	Priengprom et al., 2015
Stilbenoid	Oxyresveratrol	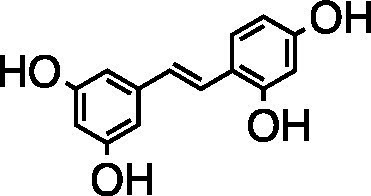	Vero Cells CC_50_ = 237.5 μg/ml HSV-1 ACVr TK-deficient IC_50_ = 25.5 ± 1.5 μg/ml HSV-1 ACVr PAAr IC_50_ = 21.7 ± 2.6 μg/ml	Inhibition of late protein production (gD, gC)	Chuanasa et al., 2008
Xanthone	Mangiferin	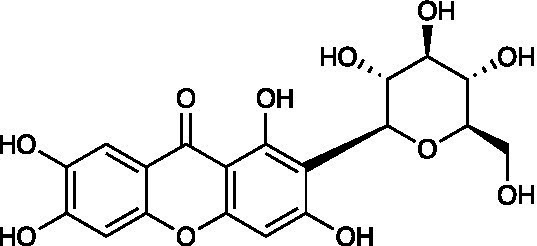	Vero Cells CC_50_ > 500 μg/ml HSV-1 ACVr AR-29 IC_50_ = 2.9 μg/ml	Virucidal effect; Inhibition of viral adsorption	Rechenchoski et al., 2020

EC_50:_ half-maximal effective concentration; CC_50:_ half-maximal cytotoxic concentration; IC_50_: half maximal inhibitory concentration; TC_50_: half toxic concentration; SI: selectivity index; SI_50_: selectivity index (SI=SI_50_); MNTC: maximum nontoxic concentration; VI: virucidal index; PI: percentage of inhibition; VII: viral inhibition index.

## Medicinal plant extracts with anti-herpes simplex virus activities

The crude hydroethanolic extract (RCE40) from the leaves of *Cecropia glaziovii* Senthl. inhibited the replication of HSV-1 ACVr strain 29R, with an EC_50_ of 40 μg/ml and a selectivity index (SI) of 50. The antiherpes properties of RCE40 might be attributed to their phenolic composition (Petronilho et al., 2012).

The water extract of *Cocos nucifera* L. husk fiber that is rich in catechin exhibited inhibitory activity against HSV-1 ACVr. The fraction exhibited higher antiviral activity than the crude extract. Mechanistically, the antiviral activity has been attributed to inactivating the extracellular virus with a vircidual effect (Esquenazi et al., 2002). Furthermore, extracts containing catechin and condensed tannins have been reported to inhibit HSV adsorption and replication (De Bruyne et al., 1999; Serkedjieva and Ivancheva, 1999).

Peppermint oil, an essential oil extracted from the leaves of *Mentha piperita* L., mainly consists of methanol (42.8%), menthone (14.6%), isomenthone (5.9%), menthylacetate (4.4%), cineole (3.8%), limonene (1.2%) and carvone (0.6%). Peppermint oil inhibited HSV-1, HSV-2, and HSV-1 ACVr infection in a dose- and time-dependent manner. It showed an obvious virucidal effect when mixed with HSV prior to infection, which implies its direct interaction with the viral envelope and glycoproteins (Schuhmacher et al., 2003). This finding suggests that peppermint oil might be used topically as a virucidal agent in the treatment of recurrent herpes infections.

Viracea, a proprietary formula, is a blend of benzalkonium chloride and phytochemicals derived from the aerial parts of *Echinacea purpurea*, which was found to have significant antiviral activity against 25 different ACV-susceptible strains (13 strains of HSV-1 and 12 strains of HSV-2) and 15 ACV-resistant strains (5 strains of HSV-1 and 10 strains of HSV-2), with a therapeutic index in the range of 50–100. Instead of benzalkonium chloride, which was speculated to be a stabilizer, the phytochemicals had anti-HSV activity after the adsorption and penetration step (Thompson, 1998).

Lamiaceae ethanolic extracts from *Prunella vulgaris* L., dried leaves of *Mentha × piperita* L., *Rosmarinus officinalis* L., and dried herbs of *Thymus vulgaris* L. inhibited the infectivity of ACV-susceptible strains and HSV-1 ACVr strain Angelotti (ACV-resistant with a single point mutant in the DNA polymerase gene) and 1246/99 (ACV-resistant patient isolate with a single point mutant in the coding sequence of the TK gene) with 50% inhibitory concentrations (IC_50_) of 0.05–0.82 μg/ml, which exerted the potential topical antiviral effect in the treatment of recurrent herpes labialis. Time-on-addition assays suggested that 80% prunella and peppermint ethanolic extracts had direct inactivation of free HSV particles and suppression of virus attachment to host cells. Additionally, the antiherpetic activity of the extracts was thought to be related to the amount and composition of phenolics in the plants (Reichling et al., 2008).

The polysaccharide from *Eucheuma gelatinae* (EGP) had comprehensive antiviral activity not only for standard experimental strains but also for clinical HSV-1 ACVr strains 106 (ACV-resistant with TK mutant), 153 (ACV-resistant with TK mutant), and Blue (ACV-resistant with TK deletion). EGP exerted its antiviral activities mainly in the early stages of HSV-1 infection, involving direct inactivation of the virions and interference in virus adsorption as a consequence of interactions between EGP and viral glycoproteins. The PCR results showed that the RNA synthesis of the HSV-1 early gene UL52 and the late gene UL27 was suppressed by EGP. In addition, the intracellular genome copy number in the EGP-treated group significantly declined, indicating that EGP inhibited viral DNA synthesis. In addition, EGP not only suppressed the synthesis of HSV-1 capsid protein VP5 but also affected cellular localization through indirect immunofluorescence and Western blot assays (Jin et al., 2015).

Essential oils from ginger (*Zingiber officinale*), thyme (*Thymus vulgaris*), hyssop (*Hyssopus officinalis*), and sandalwood (*Santalum album*) are complex mixtures of low-molecular-weight molecules and the active components of lipophilic carbohydrates. EO inhibited the infection of HSV-1 ACVr strains 1246/99, Angelotti, and 496/02 (ACV-resistant patient isolate with a single point mutant in the coding sequence of the TK gene). The essential oils prior to infection caused significant reductions in infectivity and proved their virucidal activity by adding the oils at different times during the HSV infection cycle (Schnitzler et al., 2007). In addition, the essential oil from *Salvia desoleana* Atzei & V. Picci, which contains linalyl acetate and alpha terpinyl acetate, suppressed both HSV-2 and HSV-2 ACVr in the postinfection stage. It revealed that the EO might interfere with later steps of the virus replication cycle (e.g., uncoating, genome replication, virus assembly or exit, cell-to-cell spread) by virus inactivation and time-of-addition assays (Cagno et al., 2017). The antiviral effect of EO was totally due to the active fraction SD1, which was characterized by mono- and sesquiterpene hydrocarbons. Considering the limitation that short-term systemic bioavailability of the essential oils and high effective dosage might lead to cytotoxicity, other anti-HSV agents should be added alongside topical treatment against recurrent infection.

*Acacia nilotica* (L.) has been used as a traditional healer to treat sexually transmitted infections (STIs), such as syphilis, gonorrhea, and other HIV/AIDS-related diseases (Kambizi and Afolayan, 2001; Chinsembu, 2016). The methanolic extract of *Vachellia nilotica*, known by the taxonomic synonym *A. nilotica*, exerted antiviral activity against HSV-2 and HSV-2 ACVr. The methanolic bark extract, which was mostly composed of saponins and flavonoids with traces of tannins, exerted a dual-mode of action against HSV-2 infection, which interfered with early steps of the virus replication cycle instead of targeting the cell surface, including inactivation of virus extracellular particles and inhibition of virus attachment (Donalisio et al., 2018).

*Vitex polygama* Cham. is used as a diuretic in traditional medicine. The flavonoid-rich ethyl acetate extract of *Vitex polygama* Cham. possessed potential activity against HSV-1 ACVr, the leaf extract possessed the most pronounced antiviral ability by inhibiting intracellular spread, and the fruit extract exhibited a virucidal effect. VPAF-1, the fraction further extracted with organic reagents, was proven to reduce viral propagation by preventing HSV-1 ACVr from attaching to cellular receptors (Goncalves et al., 2001).

*Ficus religiosa* L. belongs to the genus Moraceae and is used to treat diabetes, inflammation, and sexually transmitted infections (Singh et al., 2011; Choudhari et al., 2013). The water bark extract of this plant effectively exhibited a virucidal effect against both HSV-2 MS (ACV-sensitive) and HSV-2 ACVr strain replication. The chloroform extract inhibited the early stage of the HSV-2 replicative cycle and interfered with viral attachment and penetration through the time-of-addition assay and attachment assay. In addition, the chloroform extract limited the production of viral progeny based on the result of the viral yield reduction assay (Ghosh et al., 2016). These findings suggest that bioactive metabolites of *F. religiosa* were identified as natural therapeutic substances for genital herpes, especially in HSV-2 ACVr strains.

The sulfated polysaccharide fraction, which is isolated from the hot water extract of *Caulerpa racemose* (HWE), has a molecular weight of 5–10 kDa and more than two SO_3_^−^ groups for each sugar residue. HWE exhibited anti-herpetic activity against HSV-1/F, HSV-2/G, and HSV-1 ACVr strain B2006 (ACV-resistant with TK deficient) and field (ACV-resistant with TK deficient), with EC_50_ values in the range of 2.2–4.2 μg/ml, without any cytotoxic effects (Ghosh et al., 2004).

The *Prunella vulgaris* polysaccharide fraction (PPV) significantly reduced the antigen expression of both ACV-sensitive HSV and HSV-1 ACVr strain DM2.1 (ACV-resistant with TK deficiency) according to immunostaining and flow cytometric analyses of HSV-1 and HSV-2 antigens (Chiu et al., 2004). An anionic polysaccharide from *Prunella vulgaris* (PVP), which was isolated by hot water extraction, ethanol precipitation, and gel permeation column chromatography, was reported to possess anti-HSV activity against HSV-1 ACVr strain DM2.1 and PAAr (ACV-resistant with DNA polymerase mutant) and HSV-2 ACVr strain Kost (ACV-resistant with TK altered). According to the viral binding assay, PVP could compete with cell receptors to exert its inhibitory effect (Xu et al., 1999). These investigations provide the possibility that *P. vulgaris* developed as a self-applicable ointment for cold sores and genital herpes.

*Houttuynia cordata* is an herbal medicine of the family Saururaceae that shows anti-inflammatory, anticancer, and antioxidative activities (Chen et al., 2003; Lu et al., 2006), as well as antiviral activity against influenza virus, SARS-CoV, and HSV (Lau et al., 2008; Choi et al., 2009; Chen et al., 2011). The water extracts of *H. cordata* (HCWEs) showed low cytotoxicity in Vero cells and inhibited HSV-1 ACVr strain AR (ACV-resistant with TK mutant) infection. The molecular mechanisms of HCWEs involve anti-HSV activity through inhibition of viral entry, interfering with viral replication by suppressing viral late genes, and inhibiting HSV-induced NF-κB activation (Hung et al., 2015).

The study investigated the antiviral activity of the extract of *Arisaema tortuosum* (Wall.) Schott leaves, which have a medicinal history in India to treat piles, snake bites, and parasitic infections. Comparing seven fractions extracted by different solvents, chloroform extract (CE) exhibited the greatest antiviral activity against HSV-2 ACVr strains by inhibiting viral attachment and entry as well as the late steps in the replication cycle. The main components identified by HPLC-PDA-MS/MS analysis are apigenin and luteolin, which inhibit cell-to-cell virus spread and the production of viral progeny (Ritta et al., 2020).

Table 2 concluded the medicinal plant extracts mentioned in this review with their families, extract solvent, and antiviral efficacy (including the IC_50_, EC_50_, and mode of action).

## Bioactive components that show anti-herpes simplex virus activities

### Terpenes

Andrographolide and its derivatives were confirmed to have antiviral activities against HIV, HBV, and HSV by interacting with viral receptors, coreceptors, and enzymes related to viral replication (Jadhav and Karuppayil, 2021). 3,19-isopropylideneandrographolide (IPAD) is an andrographolide analog isolated from *Andrographis paniculata* Nees. IPAD exerted an inhibitory effect against both HSV wild types (HSV-1 and HSV-2) and HSV-1 ACVr strain ACGr4 (ACV-resistant with TK deficiency), dlsptk (ACV-resistant with TK deletion), and dxpIII (phosphonoacetic acid and phosphonoformate resistance). The inhibitory effects of IPAD are involved in the replication cycle by inhibiting the synthesis of viral DNA and the late protein gD. Furthermore, the synergistic effects of ACV and IPAD on HSV wild types and HSV-1 ACVr were determined by CPE reduction assay (Priengprom et al., 2015). The triterpene extract (TE) of birch bark and its major pentacyclic triterpenes (betulin, lupeol, and betulinic acid) were found to be active in suppressing HSV-1 strain KOS and two clinical isolates HSV-1 ACVr in a dose-dependent manner using plaque reduction assays. Unlike ACV, TE, and triterpenes achieved minor virucidal activity and antiviral effects in the early phase of infection (Heidary Navid et al., 2014).

Oleanolic acid, a pentacyclic triterpenoid that widely exists in natural products, possesses antitumor, anti-inflammatory, and hepatoprotective activities (Baer-Dubowska et al., 2021; Hosny et al., 2021; Zhang et al., 2021). Oleanolic acid exerted potent antiviral activity against the ACV-sensitive strain HSV-1/F and three HSV-1 ACVr strains (Blue, 106 and 153). Mechanistic studies demonstrated that oleanolic acid suppressed viral replication by downregulating the mRNA expression of the viral helicase-primase complex that is composed of UL5, UL52, and UL8. The *in vivo* study carried out on the HSV-1-infected zosteriform model suggested that oleanolic acid relieved skin lesions (Shan et al., 2021), which indicated that oleanolic acid could be a promising therapeutic target for HSV-1-related skin lesions, particularly in ACV-resistant individuals.

### Phenolics

The essential oil of *Lippia graveolens* (Mexican oregano) inhibited the infection of ACV-sensitive HSV-1 and HSV-1 ACVr strains at different time points of viral replication. The main component carvacrol exhibited more significant antiviral activity only when the cells were post-treated, with a different mode of action as essential oil (Pilau et al., 2011).

*Melissa officinalis* (lemon balm), belonging to the Lamiaceae family, is reported to exert antioxidant and antibacterial effects (Canadanović-Brunet et al., 2008). The aqueous extract of *Melissa officinalis* interfered with the early steps of virus replication against both HSV-1 KOS and two clinical isolates of HSV-1 ACVr strains. Among the phenolic compounds isolated from *Melissa* leaves, caffeic acid, p-coumaric acid, and rosmarinic acid were the main contributors by inactivating the virus and inhibiting virus attachment and penetration (Astani et al., 2014).

Ellagitannins are plant-derived polyphenol compounds with pharmacological activities, including antitumor and antibacterial activities (Ismail et al., 2016; Puljula et al., 2020). Three ellagitannins (castalagin, vescalagin, and grandinin) were proven to exhibit antiviral effects against HSV-1 ACVr strain R-100 (ACV-resistant with a TK enzyme-encoding gene mutant) and HSV-2 ACVr strain PU (ACV-resistant with TK deficiency) through the focus forming units (FFU) reduction test and CPE inhibition test. In addition, the tested ellagitannins had combined effects with ACV on the replication of HSV-1 ACVr and HSV-2 ACVr strains, and the inhibitory mechanism was different from ACV (Vilhelmova-Ilieva et al., 2014).

### Xanthones

Mangiferin, a polyphenol with a C-glycosylxanthone structure, can be discovered in mango trees (Mangifera indica) and is used as a treatment for burns and pruritus and exerts neuroprotective effects (Lee et al., 2009; Liu T. et al., 2021). Mangiferin was reported to have great *in vitro and in vivo* antiviral activity against HSV-1 ACVr strain AR-29 and HSV-1 strain KOS with low toxicity. Further mechanistic studies showed that mangiferin exerted its antiherpetic effect mainly by exerting a virucidal effect and inhibiting viral adsorption (Rechenchoski et al., 2020).

### Cardenolides

Cardiac glycosides are naturally derived compounds and are widely used in the treatment of heart failure (Kelly, 1990). After screening 65 cardenolide derivatives, the natural cardenolide compound glucoevatromonoside, which was isolated from the Brazilian cultivar of *Digitalis lanata*, exhibited potent anti-HSV activity against the HSV-1 strain KOS, HSV-2 strain 333, and HSV-1 ACVr strain 29R with low IC_50_ values of 0.13 ± 0.01, 0.04 ± 0.00, and 0.06 ± 0.01 μM, respectively. It reduced the expression of the viral proteins ICP27, UL42, gB, and gD by downregulating the K^+^ concentration into cells that is essential for viral protein synthesis, thus interfering with virus release and viral spread (Bertol et al., 2011).

### Flavonoids

Amentoflavone (AF), a naturally existing biflavonoid, was proven to exert antiviral activity against HSV-1 strain F and three HSV-1 ACVr strains (Blue, 106 and 153) through CPE and plaque assays. Mechanistically, AF completely reduced viral gene production (UL54, UL52, and UL27) and protein levels (ICP0, gD, and VP5) of HSV-1 strain F and three HSV ACVr strains. Furthermore, AF affected cofilin-mediated F-actin remodeling, which was essential for HSV-1 viral entry and reduction of viral nuclear transport (Li et al., 2019).

4’-Phenylflavone is a synthetic flavonoid that exerted antiviral activity against HSV-1 strain KOS, HSV-2 strain UW 268, and HSV-1 ACVr strain A4-3 (ACV-resistant with TK deletion) *in vitro and in vivo* by interfering with late stages in viral replication and reducing the release of progeny viruses. Synergistic therapy with 4′-phenylflavone and ACV remarkably improved the lesion scores and survival rates of HSV-infected mice, indicating the antiherpetic effect of the drug combination (Hayashi et al., 2012).

Soybean (*Glycine max*) isoflavonoids have great biological activity related to estrogenic responses. Genistein and coumestrol inhibited the infection of HSV-1 strain KOS, HSV-2 strain 333, and HSV-1 ACVr strain 29R. Coumestrol affected multiple steps in the HSV replication cycle, including adsorption and penetration, by reducing the expression of the viral proteins UL42 and gD. In comparison, genistein showed no effect on early stages but reduced the expression of viral proteins UL42, gD, and ICP27 (Argenta et al., 2015).

Baicalein is a flavonoid isolated from the root of *Scutellaria baicalensis* Georgi with biological activity against cancer and inflammatory diseases. Baicalein inhibited the replication of both HSV-1 strain F and HSV-1 ACVr strain Blue in Vero and HaCaT cells. Further mechanistic analysis elucidated that baicalein inactivated viral particles and suppressed NF-κB activation, which contributed to the protective effect of baicalein on HSV-1 infection. Furthermore, oral administration of baicalein also reduced HSV-1-induced lethality and viral loads and ameliorated symptoms in both the nose and trigeminal ganglia in an HSV-1 intranasal infection model (Luo et al., 2020).

### Proteins

MAP30 and GAP31 were derived from the Himalayan tree *Gelonium multiflorum* and Chinese bitter melon *Momordica charantia*. These two proteins were found to be effective in inhibiting the HSV-1 ACVr strain DM21 and PAAr and the HSV-2 ACVr strain Kost (ACV-resistant with TK altered) and 8,708 (ACV-resistant with TK altered) without toxicity to human embryonic cells. However, the detailed mechanism remains to be defined (Bourinbaiar and Lee-Huang, 1996).

Cyanovirin-N (CV-N), the cyanobacterial protein isolated from *Nostoc ellipsosporum*, and its two chemically modified derivatives LCV-N and PEG_10k_-LCV-N were reported to possess antiviral activity against HSV-1 ACVr strains 153, Blue, and 106. According to the WST-8 assay, PEG_10k_-LCV-N had the lowest cytotoxicity, but it showed reduced activity against ACV-resistant HSV strains compared with CV-N and LCV-N, and the detailed mechanism still needs to be explored. However, the anti-HSV-1 potency of CV-N was confirmed due to the presence of an intact sugar binding site on the B^M^ domain for viral glycoprotein interaction, which suggests that it is a viral entry inhibitor of HSV-1 and HIV (Barrientos et al., 2006; Xiong et al., 2010; Lei et al., 2019).

### Alkaloids

Caffeine inhibited the plaque formation of HSV-2 and HSV-1 ACVr strain B2006 (TK deficient) and PAAv *in vitro*. Caffeine gel treatment decelerated the development of skin lesions in mice caused by HSV-2 and reduced the virus yield of the skin infected with ACV-resistant HSV-1 strains *in vivo* (Yoshida et al., 1996).

Harringtonine (HT), a natural alkaloid isolated from *Cephalotaxus harringtonia,* possessed antiviral effects against two HSV-1 ACVr strains (Blue and 153) and three ACV-sensitive strains. A further study showed that HT reduced the expression of HVEM, thereby affecting viral membrane fusion and viral entry (Liu Y. et al., 2021).

Transdihydrolycoricidine (R430) is a lycorane-type alkaloid derivative from the Amaryllidaceae family that showed more potent antiviral activity against HSV-1 ACVr strains TK mutant and PAAr compared to ACV. R430 influenced the early stages of the HSV-1 lytic phase principally by reducing the transcription and translation of the viral immediate early (IE) gene ICP4 and DNA polymerase, which may be a consequence of upregulation of STAT3 (D'Aiuto et al., 2018).

### Steroids

*Artocarpus lakoocha* is used to treat HSV and varicella-zoster virus infection (Sangkitporn et al., 1995). Oxyresveratrol obtained from the heartwood of *Artocarpus lakoocha* Roxburgh exhibited antiherpes activity against HSV-1 strain 7401H and HSV-1 ACVr strain B2006 (ACV-resistant with TK deficiency) and PAAv by inhibiting the early gene products ICP6 and ICP8 as well as the late viral proteins gC and gD. The combination of oxyresveratrol and ACV showed a synergistic effect against HSV-1 according to isobologram analysis. In addition, 30% oxyresveratrol ointment treatment prolonged the survival rate and delayed mouse skin lesion development in HSV-1-infected mice (Chuanasa et al., 2008).

### Peptides

*Aspergillipeptide* D is a cyclic pentapeptide isolated from the fungal strain *Aspergillus* SCSIO 41501. *Aspergillipeptide* D exerted a significant antiviral effect against three HSV-1 ACVr strains, Blue, 153, and 106, by reducing the mRNA expression of UL27 and the late viral gB protein encoded by UL27. Furthermore, *Aspergillipeptide* D interfered with the expression and localization of gB in the endoplasmic reticulum and Golgi apparatus, suggesting that it mainly affected gB in viral intercellular spread instead of viral entry (Wang et al., 2020).

Table 3 summarized phytochemicals mentioned in this review with their chemical class, structure and activity against ACV-resistant HSV strains (including the IC_50_, EC_50_ and the mode of action).

## Conclusion

HSV infection is an urgent public health issue. The nucleoside analog ACV is the most effective clinical treatment that interferes with viral replication and alleviates HSV infection. However, excessive and prolonged use of ACV has led to the emergence of ACV-resistant HSV strains. Thus, it is urgent to develop an effective antiviral therapy with low toxicity to handle ACV-resistant strains (Li et al., 2022). In this review, medicinal plant extracts from 13 families, including Araceae, Asteraceae, Caulerpaceae, Cecropiaceae, Fabaceae, Lamiaceae, and Moraceae, and phytochemicals such as alkaloids, flavonoids, phenolics, terpenes and others, were concluded to exert antiviral properties against ACV-resistant HSV infection, and the mechanism of action were summarized in Figure 1. Most plant extracts that can inhibit ACV-resistant HSV infection are classified into heat-clearing and detoxifying drugs in Traditional Chinese Medicine, with a history of external use to treat sores. The majority of the active components exerted virucidal effects and viral entry inhibition, including blocking viral attachment and fusion. In addition, some phytomedicines prevented the intranuclear biosynthesis of viral proteins and cellular reinfection by progeny viruses. Nevertheless, only a few substances interfered with nuclear transportation and virion egress. In contrast to ACV, these natural products mainly interfered with the early stages of the HSV replication cycle rather than the later stages, demonstrating their potential to treat ACV-resistant HSV infection. Unfortunately, there is no candidate in clinical development or in clinical use against ACV-resistant HSV infection.

Overall, the medicinal plant and phytomedicines mentioned in this review might be promising options for treating ACV-resistant HSV infection, although further studies are needed to exploit sufficient theoretical support for antiviral therapy, such as structure optimization, the synergistic effect of medicinal plants and ACV, and confirmation of the evidence from *in vitro* and animal-based studies.

## Author contributions

LX and X-LZ equally contributed to the development of this manuscript. Z-CX assisted to revise the manuscript. All authors wrote the manuscript and designed the tables in the manuscript. All authors contributed to the article and approved the submitted version.

## Funding

This work was financially supported by the National Natural Science Foundation of China (No. 82204437), NSFC-Joint Foundation of Yunnan Province (No. U1902213), Shanghai science and technology innovation action plan (No. 22YF1445100), and Key-Area Research and Development Program of Guangdong Province (No. 2020B1111110003).

## Conflict of interest

The authors declare that the research was conducted in the absence of any commercial or financial relationships that could be construed as a potential conflict of interest.

## Publisher’s note

All claims expressed in this article are solely those of the authors and do not necessarily represent those of their affiliated organizations, or those of the publisher, the editors and the reviewers. Any product that may be evaluated in this article, or claim that may be made by its manufacturer, is not guaranteed or endorsed by the publisher.
